# KIF11 is upregulated in colorectal cancer and silencing of it impairs tumor growth and sensitizes colorectal cancer cells to oxaliplatin via p53/GSK3β signaling

**DOI:** 10.7150/jca.52103

**Published:** 2021-05-03

**Authors:** Yan Zhou, Leping Yang, Li Xiong, Kunpeng Wang, Xuyang Hou, Qinglong Li, Fanhua Kong, Xi Liu, Jun He

**Affiliations:** 1 Department of General Surgery, The Second Xiangya Hospital, Central South University, Changsha, Hunan 410011, China.; 2 Department of General Surgery, Taizhou Central Hospital, Taizhou University Hospital, Taizhou, Zhejiang 318000, China.

**Keywords:** KIF11, colorectal cancer, oxaliplatin, autophagy, GSK3β

## Abstract

Colorectal cancer (CRC) is the most frequently diagnosed cancer of the digestive tract. Chemotherapy drugs such as oxaliplatin are frequently administered to CRC patients diagnosed with advanced or metastatic disease. A deep understanding of the molecular mechanism underlying CRC tumorigenesis and identification of optimal biomarkers for estimating chemotherapy sensitivity are essential for the treatment of CRC. Numerous members of the kinesin family are dysregulated in cancers, contributing to tumorigenesis, metastasis and drug resistance. KIF11 is a key component of the bipolar spindle and is highly expressed in several cancer types. We analyzed KIF11 expression in clinical samples by Western blotting and qRT-PCR and explored its role and mechanism in CRC growth and sensitivity to oxaliplatin via detection of the phosphorylation profile of kinases and gain-and-loss-of-function assays. We found that KIF11 was upregulated in CRC tissues and was associated with advanced clinical stage and vessel invasion and that knockdown of KIF11 led to tumor growth arrest and increased sensitivity to oxaliplatin via enhanced DNA damage and apoptosis. Mechanistically, aberrantly activated p53 signaling or possibly deactivated GSK3β signaling was responsible for KIF11 knockdown-mediated effects in CRC cells. Thus, our data firmly demonstrated that KIF11 could serve as a potential oncogene and proper biomarker for assessing oxaliplatin sensitivity in CRC.

## Introduction

Although the incidence of colorectal cancer (CRC) has decreased every year since 2003, it still remains the most diagnosed cancer of the digestive system worldwide 1, 2. In China, new cases of CRC increased at a rate of approximately 1% between 2006 and 2010 3. The molecular mechanism underlying CRC progression has been greatly advanced, but surgery is the only modality for radical removal of cancer 4. Drug therapy such as oxaliplatin is preferred for advanced or metastatic disease. Despite the overall survival improvement supplied by chemotherapy, relatively limited numbers of patients benefit from this treatment, and predictive markers that suggest the individual benefit of chemotherapy are still scarce for CRC.^5^ Thus, a better understanding of the molecular mechanisms underlying tumorigenesis and screening of proper biomarkers for chemotherapy response are essential for CRC patients.

The kinesin family consists of 14 subfamilies divided by motor domain that fulfill the functions of regulators of mitosis and cytokinesis 6. The importance of kinesins in tumor development and progression is increasingly evident, and drugs that inhibit microtubule dynamics such as taxanes, vinca alkaloids or epothilones are clinically successful in various cancers 7, 8. However, a major limitation is that the microtubule-based cytoskeleton is not only required for mitosis but is also fundamental to a number of biological functions, such as axonal transport in neurons, sperm motility, or establishment of cell polarity, leading to dose-limiting side effects of these drugs 9. This situation has spurred efforts to screen and target cancer-specific kinesins for which inhibition hampers mitosis without producing significant side effects. For instance, upregulation of KIF11, a member of the kinesins required in formation of the bipolar spindle, was significantly associated with poor prognosis of oral cancer patients 10. KIF11 was also highly expressed in glioblastoma, especially in proliferating and migrating cells, and targeting of KIF11 with a small-molecule inhibitor pronouncedly inhibited initiation and self-renewal of cancer stem cells 11. Of note, no obvious neurotoxicity was observed in trials with KIF11 inhibitors such as ispinesib and AZD4877 for treatment of various solid cancers 12, 13. Similarly, CENPE, the largest kinesin, was significantly upregulated in prostate cancer, and the subtype of castration resistance further elevated its expression 14. Drugs targeting CENPE also showed promise in the treatment of pancreatic cancer, with a low number of high-grade side events 15. However, the role and molecular mechanism of these kinesins in CRC are generally less explored.

As a plus end-directed kinesin in metaphase, KIF11 is essential for cell division 16. Knockdown of KIF11 or use of specific inhibitors of KIF11 can activate the response of the spindle checkpoint, causing mitotic stress and finally leading to programmed cell death in cancer cells. In glioblastoma, upregulation of KIF11 by attenuating protein turnover plays an important role in the motility and morphogenesis of proliferating cells and affects microtubule dynamics by maintaining microtubule polymerization 11. KIF11 protein expression was improved by RNF24/40 complex-mediated histone H2B mono-ubiquitination in breast cancer, and its high expression was intimately correlated with poorer prognosis 17. A recent report also suggested that high expression of KIF11 was frequently observed in clinically more aggressive tumors in meningioma, and knockdown of KIF11 markedly inhibited cell proliferation 18. Although the role of KIF11 in tumor growth of CRC has not been thoroughly investigated 19, the evidence showing that it is required for mitosis and cell proliferation hints that it might possess a pivotal function in CRC.

Oxaliplatin is a platinum-based chemotherapy drug commonly used in CRC patients 20. Similar to the classic platinum compound cisplatin, once through the aquation process, the reactive form of platinum can bond with DNA bases to generate DNA adducts and DNA damage 21. However, poor efficacy in many patients and unavoidable side effects largely limit the application of this drug in CRC. In one trial, the result indicated that addition of oxaliplatin to combination therapy did not improve therapeutic efficacy and caused significantly increased side events in CRC patients 22. A previous report showed that downregulation of KIF11 induced chromosome instability, suggesting its potential role in oxaliplatin sensitivity 23. We sought to determine whether KIF11 silencing influences DNA status in the presence of oxaliplatin.

In this study, we demonstrated that KIF11 was upregulated in CRC and that higher expression of KIF11 was correlated with a higher grade of clinical stage. Knockdown of KIF11 markedly impaired tumor growth *in vitro* and *in vivo* and sensitized CRC cells to oxaliplatin via enhanced DNA damage and apoptosis. Mechanistically, p53 signaling was aberrantly activated, and GSK3β signaling deactivation in KIF11 silencing cell might partially explain the effects.

## Materials and methods

### Agents

Oxaliplatin and PFTα were purchased from TargetMol, America. Oxaliplatin and PFTα were dissolved in DMSO and stored in -20 °C before used.

### Human samples and CRC cell lines

This study was approved by the Ethics Committee for Human Research, Central South University and was conducted according to the approved guidelines. The patients whose tissues were used in this study supplied written informed consent in accordance with the Declaration of Helsinki. For fresh samples, 30 patients diagnosed with CRC were included, and the cancerous and paracancerous tissues were collected after surgical resection. Fresh samples were preserved at -80 °C. In addition, another cohort of 109 pairs of CRC samples was also collected for immunohistochemical staining. These samples were maintained in paraffin packaging for prolonged preservation. The CRC cell lines HT29 and HCT116 were purchased from ZSBIO, China, where cell line STR genotyping was also examined by the company. For cell culture, cells were cultured in RPMI-1640 medium supplemented with 10% FBS and 1% penicillin-streptomycin. All cells were placed in a humidified incubator with 5% CO_2_ at 37 °C.

### Cell viability/proliferation assay

Cell viability/proliferation rate was tested using the CCK-8 kit (GeneView, America). After transfection, cells were plated in 96-well plates with at least 3 replicates per group and cultured for 24 h at 37 °C. CCK-8 regents were added into each well and cultured for 3 h, followed by determination of the OD values at 450 nm using a microplate spectrophotometer (Thermo Fisher, America). All values were normalized to the control group without adding cells, and data were presented as the mean ± SD.

### Colony formation assay

After transfection, CRC cells were diluted via a dilution gradient, and finally, 5000 cells were seeded per well in 6-well plates. Cells were cultured for 7 days, followed by washing twice with PBS, fixing with 4% paraformaldehyde, and staining with 1% crystal violet staining solution (Beyotime, China). Colony images were captured by a digital camera.

### Subcutaneous xenograft model

BALB/c nude mice received humane care in compliance with the guidelines implemented at Second Xiangya Hospital, Central South University. The study was performed according to the international, national and institutional rules covering animal experiments and biodiversity rights. In brief, 2 × 10^6^ HCT116 cells transfected with KIF11 siRNA or control siRNA were subcutaneously injected into the right dorsal of 6-week-old male nude mice (n=5). After feeding for 4 weeks, mice were sacrificed, and tumors were collected.

### Western blot analysis

CRC samples and cells were lysed in RIPA buffer supplemented by protease and phosphatase inhibitors (TargetMol, America) and incubated on ice for 30 min. The supernatant was collected after discarding the sedimentation. The denatured proteins were added to the chamber for electrophoresis in SDS-PAGE and subsequently transferred onto PVDF membranes by electrotransfer. After blocking with 3% BSA for 1 h, the membranes were incubated with diluted primary antibody overnight at 4 °C. On the following day, after discarding the primary antibody, the samples were washed three times with TBST and incubated with diluted secondary antibody for 1 h at room temperature. The immune complexes were detected via an enhanced chemiluminescence system (Life Tec, America). Analysis and quantification of the bands were performed by ImageJ software (Version 11). The primary antibodies involved in this work include KIF11 (1:1000, CST, America), γH2AX (1:1000, Abclonal, China), caspase 3/cleaved caspase 3 (1:1000, CST, America), caspase 9/cleaved caspase 9 (1:1000, CST, America), Bax (1:1000, Abclonal, China), Bad (1:1000, Abclonal, China), and GAPDH (1:1000, Abclonal, China). Secondary antibodies were purchased from Abclonal, China.

### Quantitative real-time PCR (qRT-PCR)

The RNA of tissues and cells were extracted via the standard schedule, as previously described 24. In brief, TRIzol reagent was added to the samples, followed by lysing for 10 min, centrifugation at 12000 g for 15 min, and eduction with isopropanol. The RNA purity and concentration were tested using a Nanodrop 2000 spectrophotometer (Thermo Fisher, America). cDNA was synthesized using a high-capacity cDNA reverse transcription kit (Life Tec, America), according to the manufacturer's guidelines. The primers are listed as follows: KIF11, 5'-TACGACACCACAGAGGAA-3' (forward) and 5'-CCACACCAGCATCTACAG-3' (reverse). 2X Universal SYBR Green Fast qPCR mix (Abclonal, China) was used in qRT-PCR on a LightCycler 96 system (Roche, America).

### Immunohistochemical staining (IHC)

The IHC analyses of clinical samples were generally performed as previously described 24. In brief, sections with 4 μM thickness were prepared from paraffin-embedded samples, processed sequentially through deparaffinization, incubated with 3% H_2_O_2_ in the dark for 15 min, and subjected to heat-induced epitope retrieval using sodium citrate buffer (10 mM sodium citrate and 0.05% Tween 20 at pH 6.0) at 96 °C for 30 min. After three washes with PBS, the sections were incubated with rabbit anti-human KIF11 or anti-Ki67 primary antibody for 2 h. Rabbit control IgG served as the control antibody. After incubation of Solution A (ChemMateTMEnVision+/HRP) for 30 min, DAB staining and hematoxylin counterstaining were performed. The sections were dehydrated, soaked in xylene, and mounted with neutral balsam.

### Immunofluorescence

Cells were seeded in 24-well plates and cultured for 24 h, washed twice with PBS, fixed with 4% paraformaldehyde and permeabilized with 0.25% Triton X-100 (Sigma-ALDRICH, America), and 3% bovine serum albumin was used to block nonspecific antigen-antibody reaction. After incubation of primary anti-γH2AX antibody or anti-LC3 antibody overnight and washing three times with PBST, cells were incubated with donkey anti-rabbit Alexa Fluo488 (Invitrogen, America) for 1 h at room temperature. Cells were rinsed and stained with DAPI (Invitrogen, America). The immunofluorescence signal was detected by a fluorescence microscope (Olympus Inc., America).

### EdU assay

Cells were seeded in 24-well plates and cultured for 24 h. The next day, the cells were rinsed once with PBS, EdU was mixed with the culture medium, and the cells were incubated for 2 h at 37 °C and then rinsed three times with PBS. After permeabilizing with 0.25% Triton X-100 (Sigma-ALDRICH, America), cells were stained by DAPI, and images were captured by a fluorescence microscope (Olympus Inc., America).

### siRNA transfection

For transfection, the cells were seeded in 6-well plates and cultured for 24 h at 50% confluence. The siRNAs or negative controls (50 nmol) were transfected using Lipofectamine™ RNAiMAX transfection reagent (Invitrogen, America). The targeted sequences of KIF11 were GAACTTGAAACCACTCAAA. After an incubation period of 48 h, cells were prepared for functional assays.

### Flow cytometry assay

The proportion of apoptosis was detected by flow cytometry after cells were stained with Annexin V and PI. The Annexin V/PI Apoptosis Detection Kit was purchased from Yeasen, China and used according to the manufacturer's guidelines. In brief, cells in various groups were harvested, washed three times with PBS, resuspended in 1X binding buffer and stained with Annexin V and PI solution for 30 min at 37 °C without light. The cells were sent for testing via BD FACSARIA II flow cytometry (Becton Dickinson, America).

### Human phospho-kinase array

The Human Phospho-kinase Array Kit was purchased from RnDSystems, America and used according to the manufacturer's guideline. The membrane is divided into two portions (A and B) to improve sensitivity and minimize cross-reactivity. In brief, the membrane was rinsed with blocking buffer, diluted cell lysate was added, and the cells were incubated overnight at 4 °C. The membrane was washed three times with PBS, followed by incubation with the antibody cocktail for 2 h at room temperature. The membrane was subsequently washed three times, and Streptavidin-HRP was applied to each membrane, and the membranes were incubated for 30 mins. Finally, the membrane was washed three times, Chemi Reagent Mix was applied, and exposure to film was conducted.

### Statistical analysis

All statistical analyses were performed using Prism software (GraphPad Prism 6). A 2-tailed Student's t-test was used to assess significant differences between two groups. For three or more groups, one-way ANOVA was used. The threshold of the P-values was 0.05, and values less than 0.05 were considered statistically significant.

## Results

### KIF11 was upregulated in CRC and correlated with clinical stage

**Figure 1:**
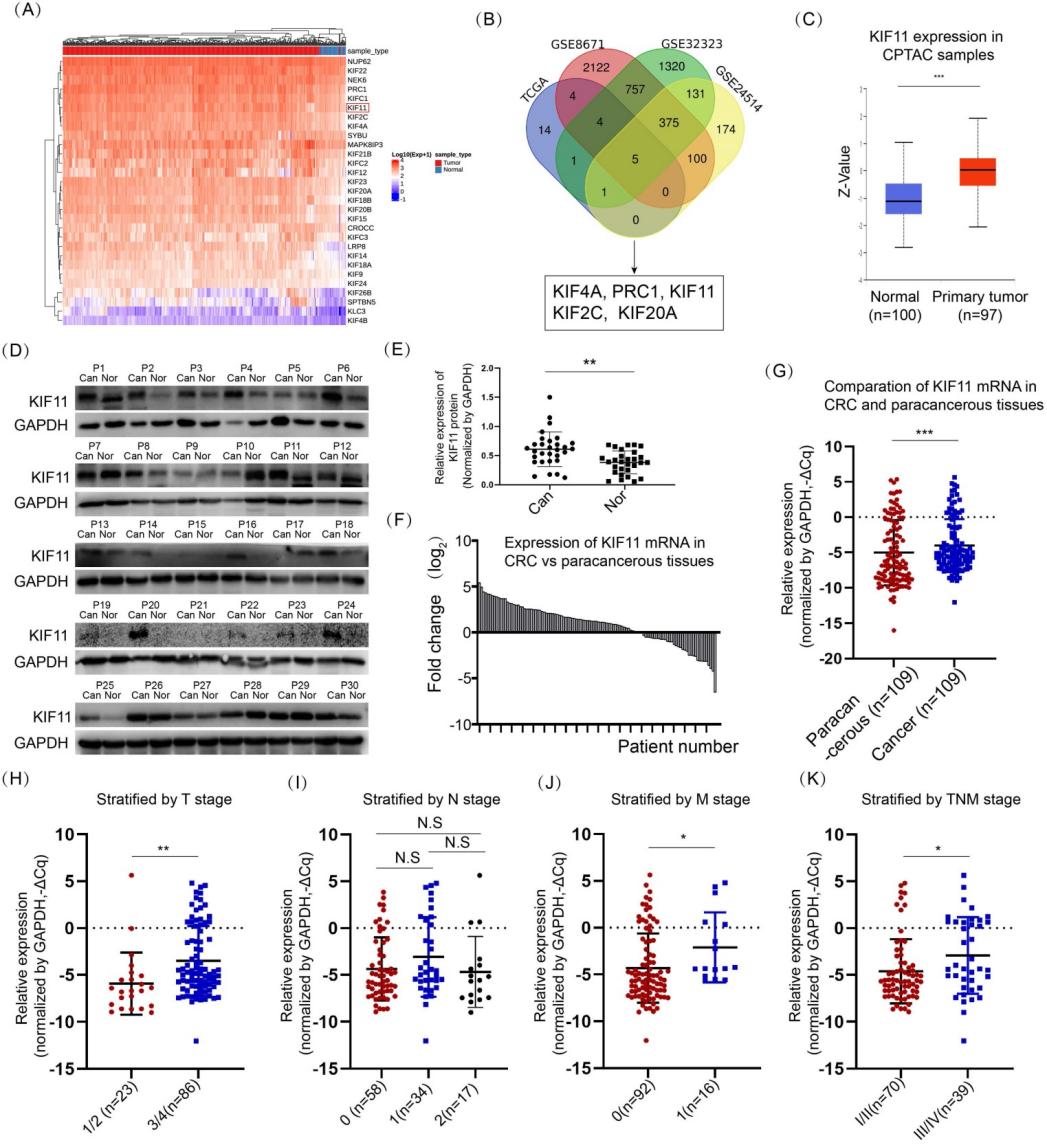
** KIF11 was upregulated in CRC compared with paracancerous tissues. (A)** The expression of 89 kinesins was acquired in datasets of the TCGA COAD (colon adenocarcinoma) program and READ (rectum adenocarcinoma) program. Among them, 29 kinesins were upregulated in colon and rectum adenocarcinoma, and the expression values in colon adenocarcinoma are presented in the heatmap. **(B)** Three datasets, GSE8671, GSE32323, and GSE24514, were used to further screen potential kinesins, resulting in five kinesins, KIF4A, PRC1, KIF11, KIF2C and KIF20A. **(C)** Expression of KIF11 protein was significantly upregulated in colon cancer compared with normal tissues using data from CPTAC. **(D)** 30 pairs of fresh samples from CRC patients were collected, and protein expression of KIF11 was tested by Western blot. Nor, normal; Can, cancer; P, patient. **(E)** KIF11 was highly expressed in fresh CRC samples compared with paracancerous tissues. **(F)** 109 pairs of CRC patients were collected, and KIF11 mRNA of CRC and paracancerous tissues were examined by qRT-PCR. KIF11 mRNA was upregulated in most pairs of patients. **(G)** Relative expression of KIF11 mRNA in paracancerous tissues and CRC tissues. ΔCq refers to the Cq of the target gene minus that of the reference gene; thus, -ΔCq represents HELLS expression level normalized by GAPDH. **(H)** Scatter plot showing KIF11 mRNA stratified by T stage; n(T1/2)=23, n(T3/4)=86. **(I)** Scatter plot showing KIF11 mRNA stratified by N stage; n(N0)=58, n(N1)=34, n(N2)=17. **(J)** Scatter plot showing KIF11 mRNA stratified by M stage; n(M0)=92, n(M1)=16. **(K)** Scatter plot showing KIF11 mRNA stratified by TNM stage; n(TNMI/II)=70, n(TNMIII/IV)=39. N.S, no significance; *P<0.05, ** P<0.01, ***P<0.001.

We queried the expression of 86 kinesins using data derived from a TCGA colon adenocarcinoma dataset and a rectum adenocarcinoma dataset. A total of 29 kinesins were upregulated and concomitantly differed in both datasets, and the result from the colon adenocarcinoma data is shown in Fig. 1A. To further determine which kinesins were most significantly dysregulated in CRC, we queried the expression of these kinesins in the other three datasets, namely, GSE8671, GSE32323 and GSE24514. GSE8671 involved 64 samples collected from 32 patients diagnosed by CRC. GSE32323 detected the global gene expression between peritumoral and tumor tissues among 17 patients. GSE24514 evaluated the gene expression profiling in 34 CRC cancers and 15 normal colonic mucosas. Five members were commonly changed, including KIF4A, PRC1, KIF11, KIF2C and KIF20A (Fig. 1B). Except for KIF11, existing reports had explored the role and the mechanism of the other 4 kinesins in CRC 8, 25-27, and therefore, we focused on KIF11. The expression level of KIF11 protein was surveyed by the CPTAC program 28, showing significant upregulation in CRC tissues compared with normal tissues (Fig. 1C). We further examined the expression level of KIF11 protein using a cohort of 30 pairs of fresh samples. As shown in Fig. 1D and E, CRC highly expressed KIF11 compared with paracancerous tissues. In another cohort of 109 CRC patients diagnosed with CRC who received surgery, RNA of CRC and paracancerous tissues were extracted, and KIF11 mRNA was detected. As shown in Fig. 1F and G, CRC significantly upregulated KIF11 mRNA compared with paracancerous tissues.

We also explored the clinicopathological significance of KIF11 in CRC based on mRNA expression data. Higher grades of T stage, M stage, and TNM stage were well correlated with higher expression of KIF11 (Fig. 1H, J and K), whereas no significant association of N stage with KIF11 expression was observed (Fig. 1I). We also interrogated the expression of KIF11 with other clinical parameters, including age, gender, invasion into vessel, invasion into nerve, pathological grade, Ki67 level and plasmic CEA level in CRC (Table 1). Higher expression of KIF11 was associated with invasion into vessel and stronger Ki67 staining. Thus, these data firmly demonstrated that KIF11 was highly expressed in CRC, which was associated with the clinicopathological parameters of CRC patients.

### Knockdown of KIF11 attenuated CRC growth *in vitro* and *in vivo*

**Figure 2:**
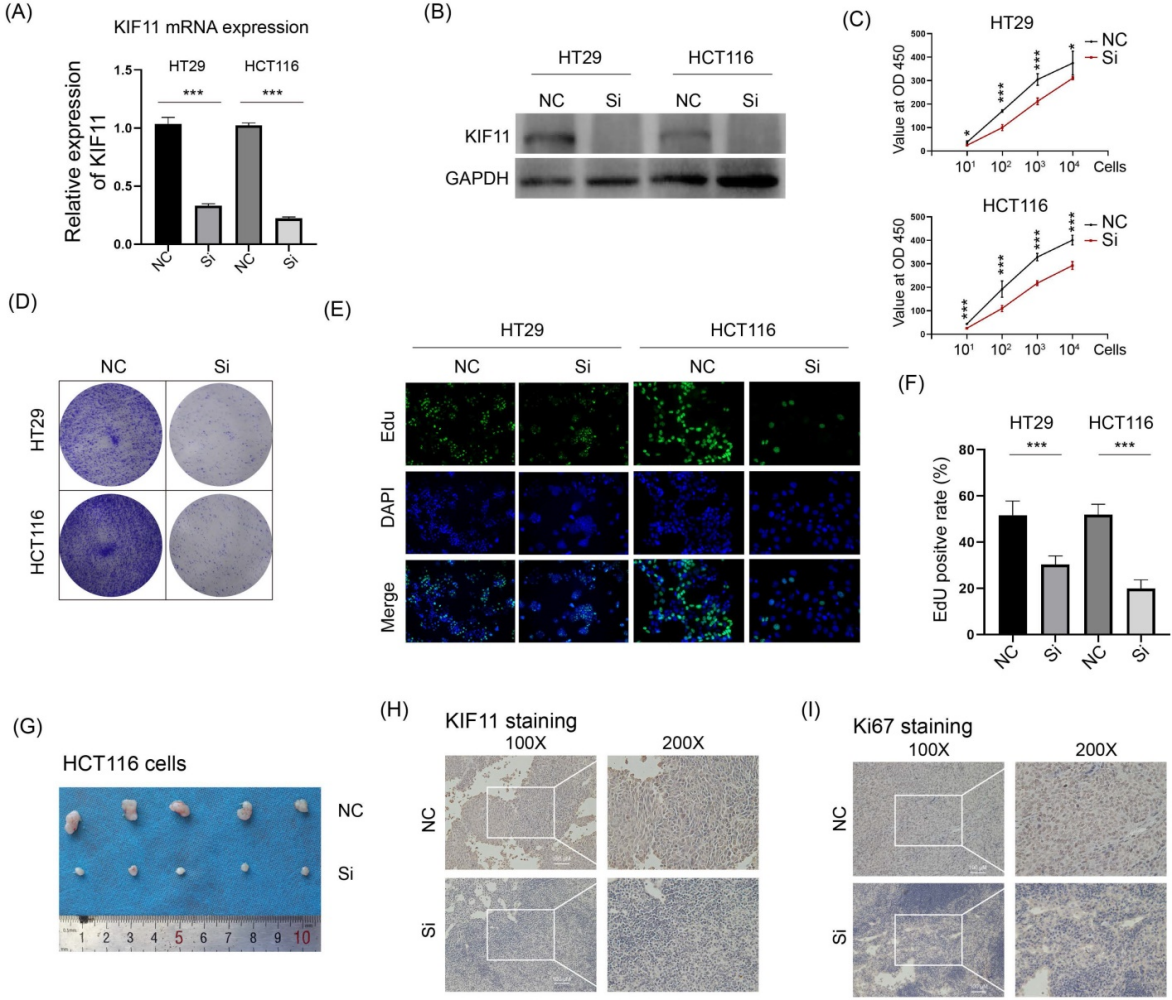
** Knockdown of KIF11 impaired CRC growth *in vitro* and *in vivo*. (A-B)** siRNA was used to inhibit KIF11 expression in HT29 and HCT116 cells, as verified by qRT-PCR and Western blot. **(C)** CRC cells were seeded at 10, 10^2^, 10^3^, and 10^4^ per well, and CCK-8 was used to detect cell numbers after 48 h incubation. Knockdown of KIF11 significantly reduced cell proliferation. **(D)** The colony formation assay was performed to determine the colony formation ability. Knockdown of KIF11 reduced colony formation in HT29 and HCT116 cells. **(E-F)** The EdU assay showed that knockdown of KIF11 significantly inhibited cell proliferation in HT29 and HCT116 cells. **(G)** HCT116 cells transfected by siRNA or control were subcutaneously injected into nude mice. After 4 weeks, mice were sacrificed and tumors were collected. **(H-I)** IHC staining of KIF11 and Ki67 demonstrated that KIF11 and Ki67 were downregulated in the siRNA group compared with the control group. NC, negative control; Si, KIF11 siRNA; *P<0.05, ***P<0.001.

As a kinesin for bipolar spindle formation, KIF11 is essential for cell division, and thus we wanted to explore whether KIF11 possesses an oncogenic function in CRC. KIF11 mRNA and protein were downregulated by siRNA in two CRC cell lines HT29 and HCT116 (Fig. 2A and B). The CCK-8 assay and colony formation assay were performed to determine whether downregulation of KIF11 could influence cell growth *in vitro*. The results showed that knockdown of KIF11 remarkably decreased the cell proliferation rate and the reduced colony formation ability (Fig. 2C and C). Consistently, the EdU assay also demonstrated that silencing of KIF11 greatly reduced cell proliferation in HT29 and HCT116. Furthermore, HCT116 cells transfected with KIF11 siRNA were subcutaneously injected into nude mice. Compared with that of the control group, the tumor volume of the siRNA group was significantly reduced (Fig. 2D). The samples were used in IHC staining of KIF11 and Ki67, which showed that KIF11 was downregulated in the siRNA group and correlated with poor Ki67 staining (Fig. 2E and F). Thus, these data demonstrated that knockdown of KIF11 significantly impaired cell growth *in vitro* and *in vivo*, which unveiled an oncogenic role of KIF11 in CRC.

### Knockdown of KIF11 sensitized CRC cells to oxaliplatin via promotion of DNA damage and apoptosis

**Figure 3:**
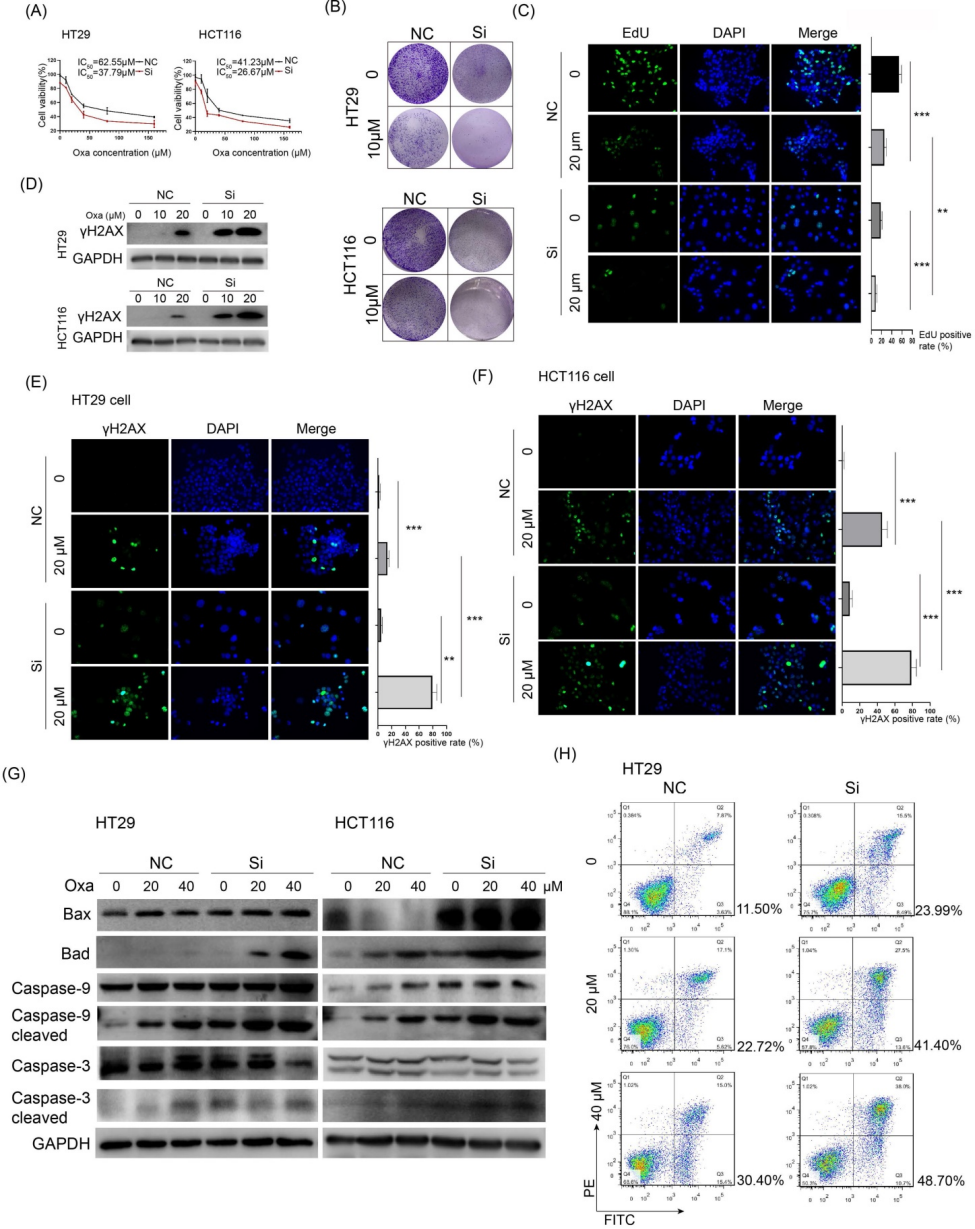
** Knockdown of KIF11 sensitized CRC cells to oxaliplatin via enhanced DNA damage and apoptosis. (A)** Cell viability was detected by CCK-8 assay in CRC cells treated with various concentrations of oxaliplatin for 24 h. The IC_50_ values of the NC and siRNA group were 65.55 and 37.79 µM, respectively, in HT29 cells and 41.23 and 26.67 µM, respectively, in HCT116 cells. **(B)** 5000 cells per well were seeded and cultured for 4 days, subsequently treated with oxaliplatin, and cultured for another 3 days. Knockdown of KIF11 markedly inhibited colony formation of HT29 and HCT116 cells treated with 10 µM oxaliplatin. **(C)** Knockdown of KIF11 reduced cell proliferation of HT29 and HCT116 cells treated with 20 µM oxaliplatin, as demonstrated by EdU assay. **(D-F)** γh2AX served as a sensitive marker for DNA damage and was detected by Western blot and immunofluorescence. Obviously, knockdown of KIF11 led to much enhanced expression of γh2AX in both cells. **(G)** Detection and comparison of the expression of caspase 3, cleaved caspase 3, caspase 9, and cleaved caspase 9 as well as Bax and Bad between the KIF11 siRNA group and control group treated with 0, 20, or 40 µM oxaliplatin. These pro-apoptosis proteins were concomitantly upregulated in the KIF11 siRNA group compared with the control group, indicating that knockdown of KIF11 facilitated apoptosis induced by oxaliplatin. **(H)** The proportion of apoptosis was quantitatively examined by flow cytometry. The apoptosis rates were 41.40% and 48.70% when treated by 20 and 40 µM oxaliplatin in the siRNA group vs. 22.72% and 30.40% in the control group. Oxa, oxaliplatin; NC, negative control; Si, siRNA; **P<0.01, ***P<0.001.

KIF11 targeting agents showed high efficacy in mouse models of cancer, and the kinesin family also suggested promise in combination therapy or in overcoming therapy resistance in cancer.^6^ We wanted to know whether interfering KIF11 would influence the sensitivity of CRC cells to oxaliplatin, a commonly used chemotherapy drug for CRC patients. CCK-8 assay showed that KIF11 siRNA remarkably decreased the IC_50_ values of oxaliplatin in HT29 and HCT116 cells compared with the control group (37.79 vs. 62.55 μM in HT29 and 26.67 vs. 41.23 μM in HCT116, Fig. 3A). The colony formation assay showed that KIF11 siRNA greatly decreased colony formation and further attenuated it in the presence of oxaliplatin in both cells (Fig. 3B). The observation was also verified by the EdU assay, and compared with the control group, KIF11 siRNA significantly reduced cell proliferation in CRC cells treated with oxaliplatin (Fig. 3C). Thus, knockdown of KIF11 significantly improved the sensitivity of CRC cells to oxaliplatin.

Platinum drugs such as oxaliplatin acquire a high affinity to DNA after they are aquated, leading to DNA damage and activating DNA damage response. We checked whether KIF11 could influence DNA damage of oxaliplatin in CRC cells. The γh2AX phosphorylation form of H2AX rapidly bonded with the double strand break (DSB) of DNA, which generally served as a sensitive marker for DNA damage. Western blot analysis showed much enhanced expression of γh2AX in KIF11 siRNA cells compared with the control group when treated by oxaliplatin (Fig. 3D). A similar result was also shown by immunofluorescence assay in which a significantly stronger signal of γh2AX was observed in KIF11 siRNA cells compared with the control group in both cells (Fig. 3E and F). We detected the expression of several pro-apoptosis proteins in CRC cells treated with various concentrations of oxaliplatin. As shown in Fig. 3G, the mitochondrial cell death pathway was activated by oxaliplatin with a dose-dependent pattern reflected by the sequentially elevated expression of Bax, Bad, cleaved caspase 3 and cleaved caspase 9 in HT29 and HCT116 cells. Importantly, with the same concentration, KIF11 siRNA led to increased pro-apoptosis proteins compared with the control group in both cells. Of note, the proportion of the apoptosis rate was largely increased in the siRNA group compared with the control group at the same concentration of oxaliplatin (Fig. 3H). Collectively, these data firmly verified that interfering KIF11 significantly sensitized CRC cells to oxaliplatin via promotion of DNA damage and apoptosis.

### p53 signaling was aberrantly activated upon KIF11 knockdown, which was responsible for enhanced sensitivity to oxaliplatin

**Figure 4:**
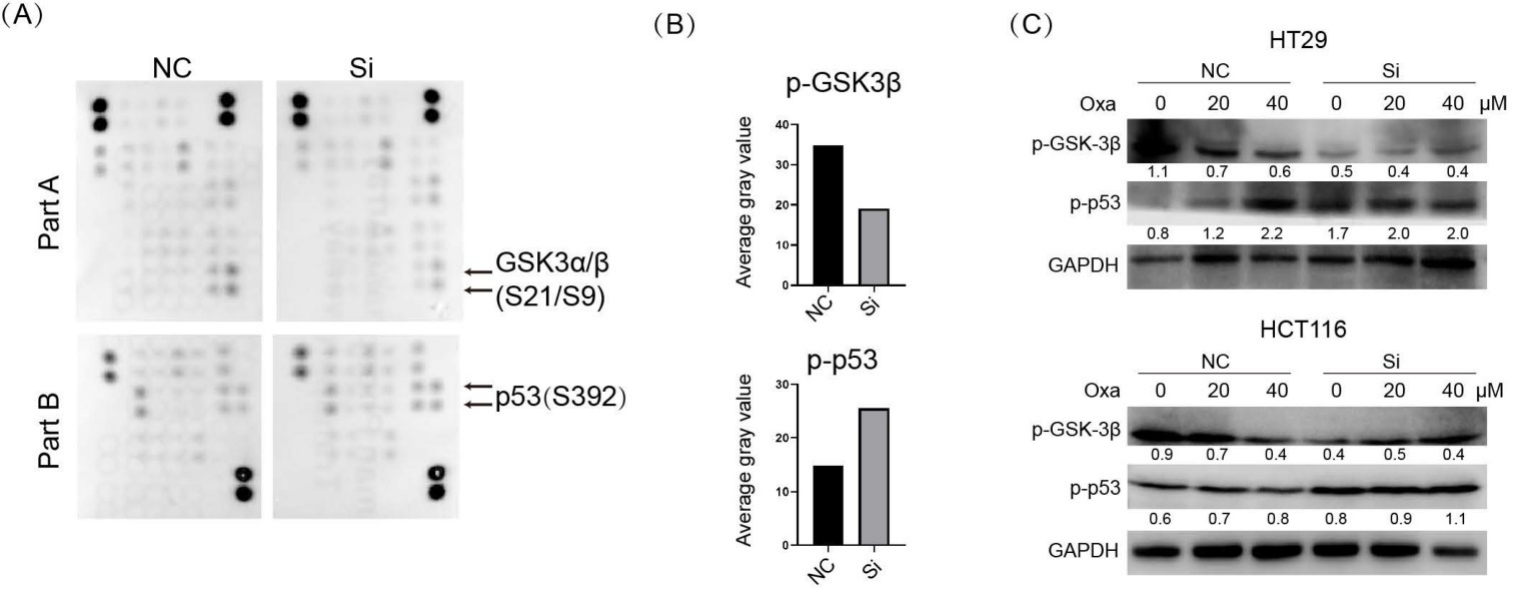
** Knockdown of KIF11 activated p53 signaling and deactivated GSK3β signaling. (A)** The Human Phospho-kinase Array Kit was used to simultaneously detect the relative levels of phosphorylation of 43 kinases. We detected the phosphorylation profiles of the KIF11 siRNA group and control group. **(B)** Histogram showing that p-p53 (S392) was upregulated and p-GSK3β (S9) was downregulated in siRNA group. **(C)** Detection of p-GSK-3β and p-p53 expression in HT29 and HCT116. p-p53 was upregulated in a dose-dependent pattern, and p-GSK-3β was decreased in a dose-dependent pattern. The siRNA group further increased p-p53 signaling compared with the control group at the same concentration of oxaliplatin, whereas p-GSK-3β further decreased in the siRNA group. NC, negative control; Si, siRNA; Oxa, oxaliplatin.

**Figure 5:**
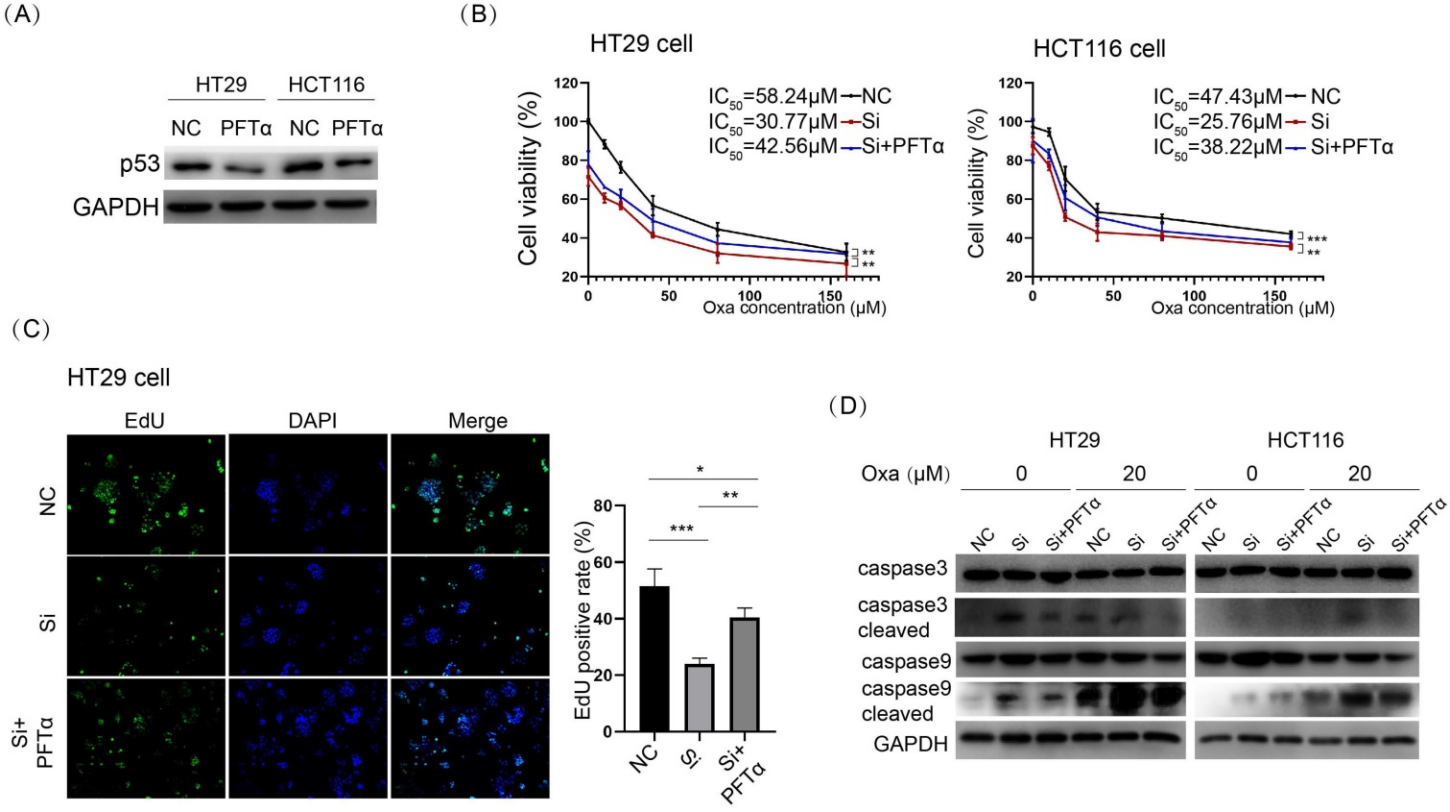
** p53 inhibitor abolished KIF11 knockdown-mediated sensitization to oxaliplatin in CRC cells. (A)** PFTα was used to inhibit p53 expression at 10 µM, as demonstrated by Western blot. (B) Treatment of PFTα significantly improved cell viability in KIF11 knockdown cells. The IC_50_ values were 58.24 µM, 30.77 µM, and 42.56 µM, respectively, in the NC group, siRNA group, and siRNA+ PFTα group in HT29 cells, and in HCT116, the values were 47.43 µM, 25.76 µM, and 38.22 µM, respectively. (C) The EdU assay confirmed that PFTα rescued cell proliferation in KIF11 knockdown cells. (D) Western blot analysis demonstrated that PFTα decreased the expression of cleaved caspase 3 and cleaved caspase 9 in KIF11 knockdown cells, indicating that PFTα rescued KIF11 knockdown-mediated apoptosis. NC, negative control; Si, siRNA; Oxa, oxaliplatin. *P<0.05, **P<0.01, ***P<0.001.

In addition to an essential role in mitotic spindle formation, the kinesins family was also found to play a noncanonical function in cancer 6. We examined the potential influence of KIF11 on the cellular signal pathways in HT29 cells. We used the Human Phospho-Kinase Array Kit to determine the level of protein phosphorylation of 43 kinases in parallel. The result showed substantially elevated p-p53 and a conversely decreased p-GSK3 signal upon KIF siRNA in HT29 cells (Fig. 4A and B). We verified the result from the kinase array via Western blot in HT29 and HCT116 cells (Fig. 4C). Moreover, p-p53 was upregulated with a concentration-based pattern upon oxaliplatin treatment, and knockdown of KIF11 led to consistent upregulation of p-p53 at the same concentration of drug. We interrogated whether activated p53 signaling was responsible for KIF11 knockdown-mediated sensitization to oxaliplatin. PFTα, a p53 inhibitor, was used to inhibit p53 expression, as demonstrated by Western blot (Fig. 5A). The cell viability was significantly rescued by PFTα treatment in the KIF11 siRNA group with various concentrations of oxaliplatin in HT29 and HCT116 cells (Fig. 5B). Furthermore, PFTα could also partially rescue proliferation arrest in KIF11 knockdown cells (Fig. 5C). As expected, apoptosis was reduced in KIF11 knockdown cells with cotreatment of PFTα and oxaliplatin, as reflected by the decreased expression of cleaved caspase 3 and cleaved caspase 9 in both cells (Fig. 5D). Thus, p53 signaling was activated by KIF11 knockdown and served as a key downstream mediator of KIF11 that was responsible for the enhanced sensitivity to oxaliplatin in CRC cells.

### GSK3β signaling was inhibited upon KIF11 knockdown, which might be the reason for enhanced autophagy in CRC cells

**Figure 6:**
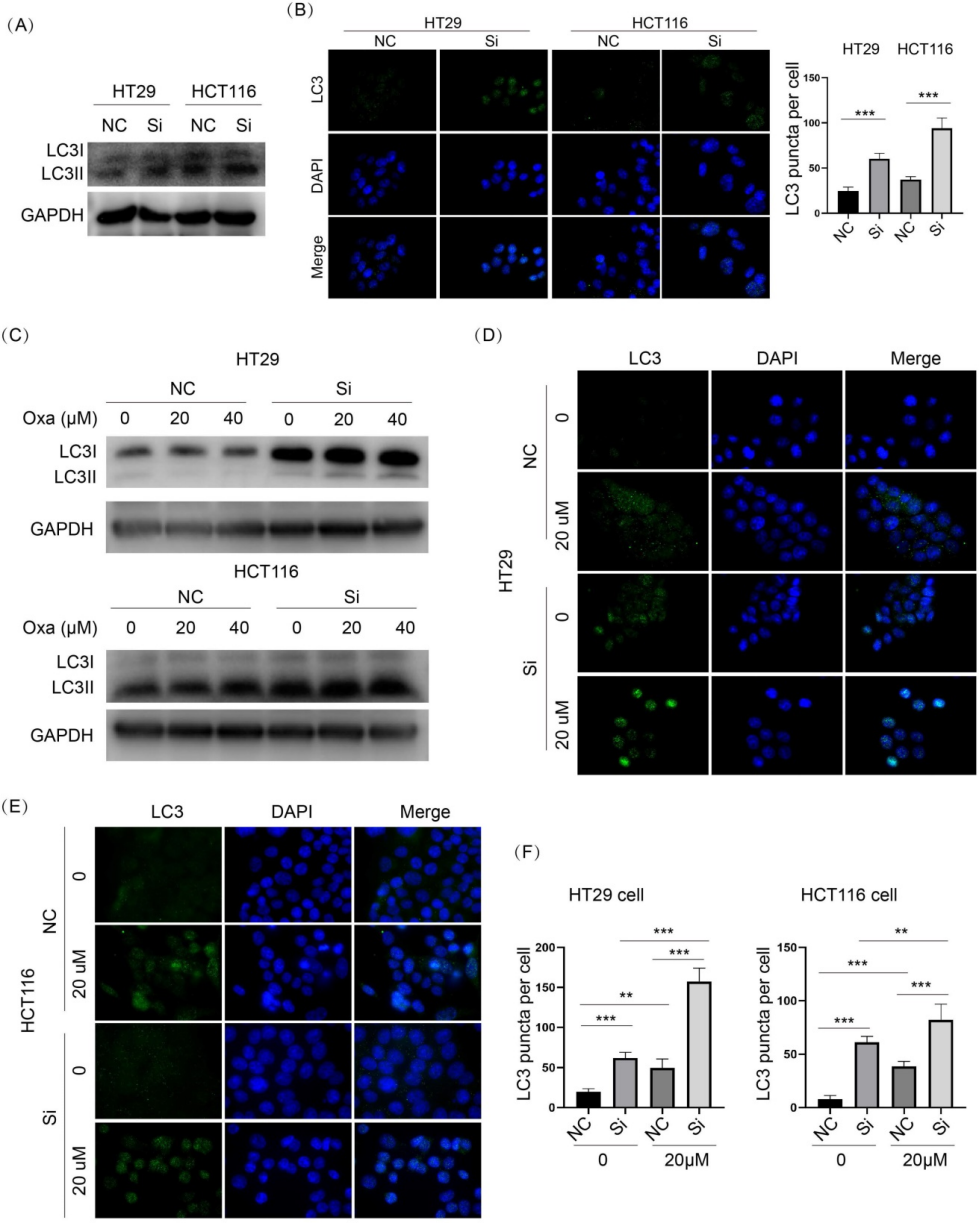
** Knockdown of KIF11 activated autophagy with or without oxaliplatin treatment in CRC cells. (A)** LC3 was a marker of autophagy. LC3II was markedly upregulated in KIF11 knockdown cells. **(B)** LC3 puncta were detected by immunofluorescence. The number of LC3 puncta was significantly higher than in than the NC groups. **(C)** In the presence of oxaliplatin, KIF11 knockdown cells expressed more LC3II than the NC groups at the same concentration. **(D-F)** The numbers of LC3 puncta were significantly increased in KIF11 knockdown cells compared with the NC groups at the same centration of oxaliplatin. NC, negative control; Si, siRNA; Oxa, oxaliplatin. **P<0.01, ***P<0.001.

As shown in Fig. 4A-C, p-GSK3β was obviously downregulated in KIF11 knockdown cells compared with control cells. Oxaliplatin treatment led to dose-dependent downregulation of p-GSK3β in HT29 and HCT116 cells. Moreover, KIF11 knockdown further decreased p-GSK3β compared with control cells treated with the same concentration of oxaliplatin. Because GSK3β is an active regulator of autophagy, we examined whether knockdown of KIF11 could influence autophagy in CRC cells. LC3II was significantly elevated in KIF11 knockdown cells compared with control cells, and the immunofluorescence assay showed an enhanced LC3 puncta signal, indicating autophagy was induced upon KIF11 knockdown (Fig. 6A and B). For the condition of oxaliplatin, the result also suggested a consistent upregulation of LC3II in KIF11 knockdown cells compared with control cells (Fig. 6C). As correlation, the number of LC3 puncta was significantly increased (Fig. 6D-F). Thus, these data indicated that autophagy was induced by KIF11 knockdown that might be derived from GSK3β deactivation in CRC cells despite the presence of oxaliplatin.

## Discussion

The kinesin family consists of more than 650 members in eukaryotes, and several members have been implicated in various cancer types.^29^ Kinesins are key components for cell division and intracellular vesicle and organelle transport and are also crucial in tumor development and drug response, which have been targeted by a variety of preclinical or clinical drugs, showing high efficiency in treatment of various cancers 30. To avoid the severe adverse effects of kinesin-targeted agents, an optimal target should be expected to be distinctly upregulated in cancers compared with normal tissues. In our study, we found that KIF11 was highly expressed in CRC compared with paracancerous tissues and was intimately correlated with clinical parameters such as T stage, TNM stage, Ki67 status and vessel invasion. These data emphasized the clinicopathological significance of KIF11 in CRC and suggested its potential role in tumorigenesis.

The canonical functions of the kinesin family, such as transport of organelles and vesicles and especially in mitosis, highlight their potential role in tumorigenesis 31. Aberrant upregulation of kinesin family members could lead to uncontrolled tumor growth, and hence mitotic kinesin inhibitors such as KIF11-targeted inhibitors and CENPE-targeted inhibitors have attracted great attention in drug development 6. For instance, it was found that KIF14 was significantly upregulated by more than 10-fold in breast cancer and that higher expression successfully predicted dismal survival rates and frequent cancer recurrence 32. Kinesis member PRC1 was upregulated in hepatocellular carcinoma compared with normal liver or paracancerous tissues and was positively associated with vascular invasion and high grade of cancer 33. In CRC, a previous report showed that KIF20A mRNA and protein were highly expressed compared with noncancer tissues and could also serve as a potential predictor of survival 26. Functionally, mounting studies revealed that the function of kinesins relied on the transportation and mitosis capabilities. KIF4A can tether Rap GTPase Interactor to microtubules, resulting in integrin activation and alteration in migration and invasion of breast cancer 34. Silencing of KIF11 led to marked mitotic arrest with an observation of the mono-astral spindle phenotype and inhibition of tumor proliferation and growth 35. In our analysis, we found that KIF11 was among the most frequently dysregulated kinesins in CRC, and its knockdown markedly impaired cell proliferation *in vitro* and tumor growth *in vivo*. Furthermore, the noncanonical roles of kinesins were identified in a variety of cancers. PRC1 bonded with the β-catenin destruction complex to improve β-catenin release from adenomatous polyposis coli complex and maintained the activity of Wnt/β-catenin signaling in hepatocellular carcinoma 33. High expression of KIF20A was essential for regulation of JAK/STAT3 signaling in CRC 26. For the first time, we demonstrated that knockdown of KIF11 activated p53 signaling, which was responsible for KIF11 knockdown-mediated sensitization to oxaliplatin in CRC. However, the precise molecular linkage between KIF11 and p53/GSK3β remains to be elucidated.

Several members of the kinesin family were verified as regulators of chemotherapy sensitivity and resistance. Overexpression of KIF5A, KIF1A or KIFC3 markedly decreased free tubulin derived from the stabilizing effect of docetaxel and hence improved drug resistance 36. Mutations in the ATP binding domain of these kinesin could abolish resistance.^36^ High expression of KIFC3 or KIF5A was suggested to predict the docetaxel response in patients of breast cancer 37. Ectopic expression of KIF20A promoted chemoresistance of CRC cells to oxaliplatin or 5-FU treatment by decreasing apoptosis 37 and also indicated that expression of KIF4A, KIF15, KIF20A and KIF23 was correlated with prognosis in breast cancer. Knockdown of these kinesins markedly promoted apoptosis induced by tamoxifen, a targeted drug 38. Of note, several KIF11-targeted drugs were examined under Phase I or Phase II clinical trials, showing moderate success as a monotherapy 12, 35. Moreover, an inhibitor of aurora A kinase could promote mono-astral spindle formation and mitotic catastrophe caused by KIF11 inhibitor, leading to apoptosis and tackling the potential resistance of the KIF11 inhibitor in cancer cells 39. In our work, we supplied direct evidence demonstrating that knockdown of KIF11 significantly improved the chemotherapy sensitivity of CRC to oxaliplatin, a commonly used chemotherapeutic drug, via enhanced DNA damage and apoptosis. In addition, it was implicated that GSK3β could positively regulate DNA repair pathway through various mechanisms, and GSK3β was also involved in the inhibition of apoptosis in cancers upon drugs 40. A thorough understanding of the cross-talk among KIF11, GSK3β, and apoptosis is needed in the future study. Nevertheless, it could be beneficial to use KIF11 as an alternative biomarker of oxaliplatin application or in combination therapy of oxaliplatin with a KIF11 inhibitor for CRC patients.

The relationship between autophagy and kinesin was largely elusive, although certain reports suggested its role in the late stage of autophagy. KIF1A was essential for local dispersion of autophagosomes in the neuron via regulation of spatial selectivity of ATG-9, a protein functioning in autophagosome biogenesis 41. KIF5B was required for autophagic lysosome reformation, a process important in maintaining lysosome homeostasis through binding with PtdIns 4, 5 P_2_ in a clathrin-dependent manner 42. We observed that knockdown of KIF11 remarkably induced autophagy and further evoked it in the presence of oxaliplatin in CRC cells. It should be noted that we did not perform direct analysis of the molecular linkage between KIF11 and autophagic flux, and thus the possibility that KIF11 knockdown-mediated DNA instability was responsible for activating autophagy rather than deactivation of GSK3β signaling could not been excluded 23, 43. Of note, autophagy was identified as a double-edged sword in the response to chemotherapy because autophagy might serve as a pathway of programmed cell death or other anti-cell death by recycling of cell components in a context-dependent pattern 44-46. Thus, whether enhanced autophagy by KIF11 knockdown could facilitate cell death or supply survival opportunity for CRC cells when treated by oxaliplatin remains as an unknown.

In closing, our study offered evidence demonstrating that KIF11 is highly expressed in CRC, correlating with certain clinical parameters, and that knockdown of KIF11 markedly impaired tumor growth and sensitized CRC cells to oxaliplatin treatment via activation of p53 signaling and deactivation of GSK3β signaling. However, a thorough exploration of the molecular mechanism underlying KIF11-mediated regulation of p53/GSK3β signaling and the effect of aberrant induction of autophagy on the efficiency of oxaliplatin is needed in future research.

## Figures and Tables

**Table 1:** The expression of KIF11 mRNA correlated with other clinical parameters in CRC

Parameters	KIF11 mRNA expression (-ΔCq)	Cases (n)	*P* value
**Age**			
≤50 yr	-4.034	18	0.9763
>50 yr	-4.005	91	
**Gender**			
Male	-4.096	75	0.7242
Female	-3.820	34	
**Invasion into vessel**			
No	-4.294	96	0.0314
Yes	-1.913	13	
**Invasion into nerve**			
No	-3.803	94	0.1507
Yes	-5.307	15	
**Pathological grade**			
Low	-4.098	46	0.1337
Median	-4.247	55	
High	-1.866	8	
**Ki 67 status**			
<50%	-5.154	32	0.0249
≥50%	-3.308	52	
**Plasmic CEA level**			
≤10 ng/ml	-4.154	76	0.4517
>10 ng/ml	-3.337	15	
